# 25-Hydroxycholecalciferol Concentration Is Associated with Protein Loss and Serum Albumin Level during the Acute Phase of Burn Injury

**DOI:** 10.3390/nu12092780

**Published:** 2020-09-11

**Authors:** Andrzej Krajewski, Krzysztof Piorun, Dominika Maciejewska-Markiewicz, Marta Markowska, Karolina Skonieczna-Żydecka, Ewa Stachowska, Zofia Polakowska, Maciej Mazurek, Małgorzata Szczuko

**Affiliations:** 1 West Pomeranian Center of Treating Severe Burns and Plastic Surgery in Gryfice, 72-300 Gryfice, Poland; krajewski1407@gmail.com (A.K.); krzysztof.piorun@gmail.com (K.P.); maciek.j.mazurek@gmail.com (M.M.); 2 Department of Human Nutrition and Metabolomics, Pomeranian Medical University in Szczecin, 71-460, Szczecin, Poland; karzyd@pum.edu.pl (K.S.-Ż.); ewast@pum.edu.pl (E.S.); malgorzata.szczuko@pum.edu.pl (M.S.); 3 Clinic of Plastic, Endocrine and General Surgery, Pomeranian Medical University in Szczecin, 72-010 Police, Poland; markowskamh@gmail.com; 4 Department of Dermatology and Venereology, Pomeranian Medical University in Szczecin, 71-010 Police, Poland; zpolakowska@gmail.com

**Keywords:** vitamin D, burns, albumin, total protein, burn body surface

## Abstract

Background: Burned patients have an increased need for vitamin D supply related to the maintenance of calcium–phosphate homeostasis and the regulation of cell proliferation/differentiation. This study aimed to analyze the concentration of 25-hydroxycholecalciferol and its relationship with severe condition after burn injury. Methods: 126 patients were enrolled in the study. Patients were qualified due to thermal burns—over 10% of total body surface area. On the day of admission, the following parameters were assessed: 25-hydroxycholecalciferol concentration, total protein concentration, albumin concentration, aspartate transaminase activity, alanine transaminase activity, albumin concentration, creatinine concentration, c-reactive protein concentration, procalcitonin concentration, and interleukin-6 concentration. Results: Almost all patients (92%) in the study group had an improper level of vitamin D (<30 ng/mL), with the average of 11.6 ± 10.7 ng/mL; 17.5% of patients had levels of vitamin D below the limit of determination—under 3 ng/mL. The study showed that there are several factors which correlated with vitamin D concentration during the acute phase of burn injury, including: total protein (*r* = 0.42, *p* < 0.01), albumin, (*r* = 0.62, *p* < 0.01), percentage of body burns (*r* = 0.36, *p* < 0.05), aspartate aminotransferase (*r* = 0.21, *p* < 0.05), and c-reactive protein (*r* = 0.22, *p* < 0.05). We did not find any significant correlation between vitamin D concentration and body mass index. Conclusions: The burn injury has an enormous impact on the metabolism and the risk factors of the deficiency for the general population (BMI) have an effect on burned patients. Our study showed that concentration of 25-hydroxycholecalciferol is strongly correlated with serum albumin level, even more than total burn surface area and burn degrees as expected. We suspect that increased supplementation of vitamin D should be based on albumin level and last until albumin levels are balanced.

## 1. Introduction

Burns are one of the most serious injuries, which often include multi-organ dysfunction. Every year, about 1% of Polish people (both children and adults) suffer from various types of burns [1]. The stress and metabolic response associated with burn injury are linked to bone demineralization. These specific conditions promote large production of glucocorticoids that decrease the number of osteoblasts and block osteoclastogenesis. Moreover, interleukin (IL) 1-β and IL-6, produced in inflammatory conditions, increase osteoclastogenic bone resorption which leads to bone loss [2]. Due to such large metabolic changes, burned patients have an increased need for vitamin D supply related to the maintenance of calcium–phosphate homeostasis [3] and the regulation of cell proliferation/differentiation [4]. Extensive burns can lead to the dysfunction of many organs, which have a big impact on vitamin D biotransformation. Liver and kidney failures are responsible for insufficient conversion of cholekalcyferol to its active metabolites. Liver dysfunction may also result in impaired production of vitamin D-binding protein [4]. Vitamin D plays a very important role in the healing of dermal wounds. In vitro studies have shown that 25-hydroxycholecalciferol had a positive effect on the regulation of the transforming growth factor beta (TGFβ). TGFβ affects many processes related to the development and regulation of cell growth, such as wound healing and scar formation. The role of the beta-transforming agent in the treatment of thermal injury wounds can be associated with the stimulation of fibroblast proliferation, myofibroblast differentiation, and collagen synthesis. Vitamin D deficiency accompanying patients after extensive burns may have a negative impact on the healing process and prolong treatment and convalescence [5].

The aim of our study was to analyze the level of vitamin D and its relationship with severe condition during the acute phase of burn injury.

## 2. Materials and Methods

### 2.1. Patients

One hundred twenty-six patients with burn injuries were enrolled in the study. Participants were patients of the Western Pomeranian Center for the Treatment of Burns Injuries and Plastic Surgery in Poland. Patients were qualified due to thermal burns—over 10% of the total body surface area (TBSA); 88% of patients involved in the project meet the major burn criteria. According to the classification used in our hospital, major burn needs to include: ≥25% TBSA, or ≥20% in adults over 40 years old, or ≥10% TBSA with full-thickness burn, or all burn injuries complicated by major trauma/inhalation injury; 12% of patients met the moderate burn criteria (10–20% partial-thickness burn). According to their medical history from admission to the unit, none of the included patients suffered from chronic kidney disease. The protocol used in our hospital includes no albumin administration during the first 24 h of burn injury. Instead of albumin patients were given Ringer’s lactate and fresh frozen plasma. The protocol of the study has been accepted by the local bioethical committee at the Pomeranian Medical University in Szczecin (KB-0012/143/16). Every participant signed a consent to take part in the study and was informed about its course, benefits, and potential side effects. Patient characteristics are shown in Table 1 and Table 2.

### 2.2. Vitamin D and Other Biochemical Parameters Measurements

On the day of admission, the following parameters were assessed: 25-hydroxycalciferol concentration (vitamin D status predictor), total protein concentration, albumin concentration, aspartate transaminase activity, alanine transaminase (ALT) activity, albumin concentration, creatinine concentration, c-reactive protein (CRP) concentration, procalcitonin concentration, and IL-6 concentration. All measurements were performed in a commercial certificated laboratory in the Hospital. The 25-hydroxycholecalciferol measurement was based on validated automatic immunochemical method. Serum was used as basic material for all analysis.

### 2.3. Statistical Analysis

The statistical analysis was performed using the “R 3.0.2” program. In order to check the normal distribution, the Shapiro–Wilk test was used. The distribution did not deviate from the norm, and parametric tests were used in the calculations. The results are presented as mean values and standard deviation (SD). In order to estimate the correlations, the Pearson’s correlation test was used. To estimate the connection between burn degree and concentration of vitamin D, the Poisson regression was used. The values of *p* < 0.05 were considered as statistically significant. To control type I errors, the false discovery rate (FDR) approach was used. The calculations were performed using the p.adjust function of the stats package in R 4.0.2. Multiple regression was used to assess the relationship between albumin, total protein, and vitamin D concentration. The values being at the threshold of statistical significance were established at *p* < 0.055 and the statistical tendency from *p* = 0.06 to *p* = 0.1. In reference to the results which were not statistically significant, the abbreviation NS (not significant) was used instead of *p*.

## 3. Results

Almost all patients (92%) in the study group had an improper level of 25-hydroxycholecalciferol (<30 ng/mL), with the average of 11.6 ± 10.7 ng/mL; 17.5% of patients had a level below the limit of determination—under 3 ng/mL. Poisson regression showed that there is a statistical tendency between 25-hydroxycholecalciferol concentration and burn degree (*p* = 0.08). The average concentration in particular subgroups is shown in Table 3.

**Figure 1:**
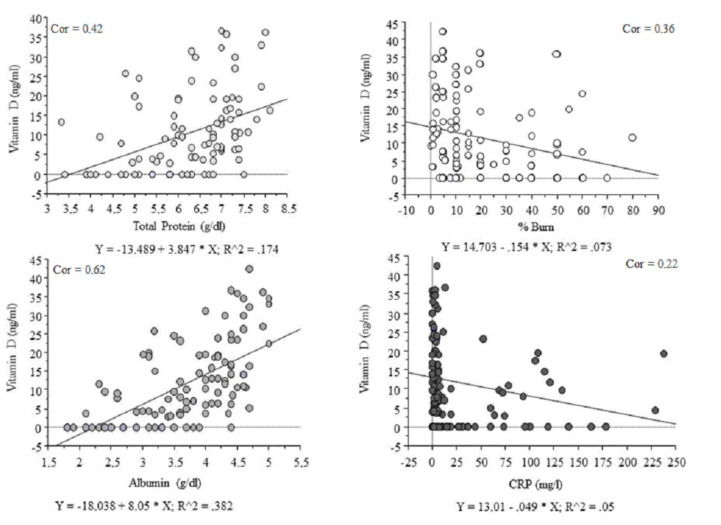
25-hydroxycholecalciferol concentration and biochemical status; CRP: c-reactive protein.

Pearson’s test showed a significant correlation between body mass index (BMI), total protein, albumin, percentage of body burns, ALT, CRP, and vitamin D concentration (Table 4). The most significant correlations are shown in Figure 1.

As we demonstrated that total protein and albumin concentration were significantly correlated (y = 2.393 + 1.069x; x2 = 0.7), we decided to conduct a multiple regression analysis to demonstrate that only albumin was significantly associated with vitamin D concentration (b = 7.9, SE = 1.68, t = 4.69, *p* < 0.0001). We also performed analysis of the correlation of percentage of body burns with albumin/protein. The results are presented in Table 5.

## 4. Discussion

Deficiencies of minerals and vitamins in burned patients are a serious clinical challenge either during hospitalization, or while in outpatient care. In many cases, the recommended supplementation is not sufficient for deficiencies and patients cannot reach the proper level of vitamin D. Dickerson et al. reported that 76% of critically ill patients after traumatic injury were vitamin D deficient and severely deficient [6]. Similar observations were made by Alizedeh et al., where 74% patient had an improper level of vitamin D [7].

Our study revealed that 92% of burned patients had an improper concentration of 25-hydroxycholecalciferol (average concentration: 11.6 ± 10.7 ng/mL) and almost 20% of them had a level below the limit of the quantification (<3 ng/mL). Płudowski et al. revealed that 89.9% of the Polish population is vitamin D-deficient, with 18.0 ± 9.6 ng/mL of average concentration of 25-hydroxycholecalciferol [8]. A lower concentration of vitamin D before admission can have a big impact on the concentration of vitamin D deficient after a burn injury and can be an additional factor of such low level during the acute phase of a burn injury.

The need of supplementation in burned patients is well known and described in many medical protocols [9]. Unfortunately, past research suggested that universal supplementation does not significantly improve concentration of Vitamin D in serum. There is no recommendation for a sufficient dose or time of increased supplementation for these group of patients [10]. Vitamin D regulates many crucial metabolic processes which are critical for burned patients’ convalescence [11] [12]. Appropriate supplementation should be implemented immediately after admission to the hospital. There is a great need to identify the factors that have the biggest influence on the concentration of vitamin D, and at the same time are analyzed during routine admission to the hospital.

There are multiple factors that are associated with low level of circulating vitamin D. Many of them are connected with poor prognosis in critically ill patients, among others: organ failure [13], sepsis, and short- or long-term mortality. Several meta-analyses have revealed that very low 25-hydroxycholecalciferol concentration is associated with the higher incidence of either infection or sepsis, and greater mortality in these groups of patients [14,15]. Our study showed that there are several factors which correlated with serum vitamin D concentration during the acute phase of burn injury (Figure 1), including: serum total protein (*r* = 0.42), serum albumin, (*r* = 0.62), percentage of body burns (*r* = 0.36), AST (*r* = 0.21), and CRP (*r* = 0.22). However, we did not see a significant relationship between BMI and concentration of 25-hydroxycholecalciferol, which are considered to be one of the most important factors of vitamin D deficiency in the general population [16]. We can assume that the burn injury has an enormous impact on the metabolism and the risk factors of the deficiency for the general population have a negligible effect on burned patients. 

Evaluation of the vitamin D level in burned patients is a difficult issue, related to the acute phase development, which is associated with decreased levels of vitamin D binding protein (VDBP). The amount of protein that can bind 25-hydroxycholecalciferol, significantly decreases, and the tested “free amount” of the active form can be falsified. Reduced protein synthesis persists for several months after burns; therefore, the results obtained can be false [17]. However, VDBP levels increase after the acute phase of thermal injury [18], but albumin concentration may recover after six months or more [19]. Due to such multifactorial problems, interpretation of 25-hydroxycholecalciferol concentration in the diagnosis of vitamin D deficiency remains challenging [20]. We have to remember that, during hospital admission, the analysis of VDBP protein or vitamin D status is not included in the standard analysis.

Our study reveals that serum protein level, mostly albumin, strongly correlated (Figure 1) with serum vitamin D status during the acute phase of burn injury. We also noticed a trend and a weaker correlation with either the burn degree or TBSA and vitamin D concentration. Therefore, these two factors are strongly associated with the plasma protein (and albumin) concentration [21]. The appropriate level of protein is essential for the maintenance of plasma colloid oncotic pressure and responsible for the transport of various substances in the blood stream including: hormones, drugs and vitamins, such as vitamin D [22]. Albumin is one of the most important proteins synthesized by the liver and has several relevant functions [23]. Because of its long half-life and the fact that serum level depends on many factors, albumin is a reliable marker of mortality and morbidity in hospitalized patients [24,25]. The acute period of burn injury is associated with severe conditions, such as: increase of free-radical oxidation and higher vascular permeability in the burned wounds, which significantly decreases the level of albumin [24]. Our study revealed that albumin strongly correlated with 25-hydroxycholecalciferol concentration (*r* = 0.62) and the multiple regression confirm that thesis. A majority (85–90%) of 25-hydroxycholecalciferol D is bound to VDBP and 10–15% to albumin [25]. The acute phase of burn injury is associated with higher vascular permeability in the burned wounds, which significantly decreases the level of all proteins, including albumin and VDBP. Yonemura et al. revealed that the active form of vitamin D (calcitriol) is associated with albumin level in patients with end-stage renal disease. Moreover, supplementation with an active form of vitamin D tends to normalize low serum albumin concentrations [26]. We can hypothesize that albumin can be a good predictor of vitamin D status, especially that the concentration of 25-hydroxycholecalciferol is not measured in standard analysis during admission to hospital [27]. However, long-term studies are needed to confirm the usefulness of albumin as a factor reflecting the need for vitamin D supplementation. It should be highlighted that burn injury decreases vitamin D synthesis in the skin, therefore patients need to be supplemented permanently [28].

## 5. Conclusions

Burn injuries have an enormous impact on the metabolism in burned patients. On the other hand, the risk factors of deficiency for the general population (e.g., BMI) have a negligible effect on burned patients. Our study shows that the concentration of 25-hydroxycholecalciferol is strongly correlated with serum albumin levels, even more than TBSA and burn degrees, as expected. Albumin can be a good predictor of vitamin D status, especially since the concentration of 25-hydroxycholecalciferol is not measured in standard analysis during admission to hospital. We presume that this direction of vitamin D diagnostic should be tested in randomized clinical trials.

## Figures and Tables

**Table 1:** Burn degree among patients enrolled in the study.

Burn Degree	Amount of Patients (*n* = 126)
I/II	9 (7%)
II	13 (10.3%)
II/III	61 (48.5%)
III	35 (27.8%)
III/IV	7 (5.6%)
IV	1 (0.8%)

**Table 2:** Patient characteristics.

Patient Characteristics	Mean	±SD
Age [years]	49.06	17.53
BMI [kg/m^2^]	24.59	3.76
Percentage of body burns [%]	21.11	20.80
Day after burn [day]	1.66	5.06
Phosphate [mmol/L]	1.18	0.22
Calcium [mmol/L]	2.29	0.26

**Table 3:** 25-hydroxycholecalciferol concentration according to burn degree.

25-Hydroxycholecalciferol Concentration [ng/mL]	Mean	±SD
Whole cohort	11.6	10.7
Superficial	18.2	13.5
Superficial partial thickness	13.08	9.9
Superficial deep dermal	11.7	13.4
Full thickness	12.8	11.7
Full thickness with catastrophic	8.45	8.3
Catastrophic	7.4	6.2

**Table 4:** Correlation between 25-hydroxycholecalciferol concentration and biochemical parameters.

25-Hydroxycholecalciferol [ng/mL] vs., *n* = 126	*r*	*p* Value	FDR * p Value
BMI [kg/m^2^]	0.18	NS	0.43
Total protein [g/dL]	0.42	*p* < 0.01	*p* < 0.01
Albumin [g/dL]	0.62	*p* < 0.01	*p* < 0.01
Percentage of body burns [%]	(−) 0.36	*p* < 0.05	0.10
AST [U/L]	(−) 0.21	*p* < 0.05	0.07
ALT [U/L]	(−) 0.08	NS	0.43
CRP [mg/L]	(−) 0.22	*p* < 0.05	0.07
IL-6 [pg/mL]	(−) 0.16	NS	0.20
Creatinine [mg/dL]	(−) 0.18	*p* < 0.055 ^#^	0.10

**^#^** the verge of significance. * false discovery rate. The false discovery rate (FDR); AST: aspartate aminotransferase; ALT: alanine transaminase; CRP: c-reactive protein; NS: not statistically significant.

**Table 5:** Correlation between percentage of body burns and protein parameters.

Percentage of Body Burns [%]	*r*	*p* Value
Total protein [g/dL]	(−) 0.58	*p* < 0.01
Albumin [g/dL]	(−) 0.62	*p* < 0.01
